# Cytotoxic and Antimicrobial Activity of Pseudopterosins and *seco*-Pseudopterosins Isolated from the Octocoral *Pseudopterogorgia elisabethae* of San Andrés and Providencia Islands (Southwest Caribbean Sea)

**DOI:** 10.3390/md9030334

**Published:** 2011-03-04

**Authors:** Hebelin Correa, Fabio Aristizabal, Carmenza Duque, Russell Kerr

**Affiliations:** 1 Departamento de Química, Universidad Nacional de Colombia, Cra. 30 N° 45-03, Bogotá D.C., Colombia; E-Mail: hcorreav@unal.edu.co (H.C.); 2 Departamento de Farmacia, Universidad Nacional de Colombia, Cra. 30 N° 45-03, Bogotá D.C., Colombia; E-Mail: faaristizabalg@unal.edu.co (F.A.); 3 Departments of Chemistry and Biomedical Sciences, University of Prince Edward Island, 550 University Avenue, C1A 4P3, Charlottetown, PEI, Canada

**Keywords:** marine natural products, pseudopterosins, *seco*-pseudopterosins, *Pseudopterogorgia elisabethae*, cytotoxic activity, antimicrobial activity

## Abstract

To expand the potential of pseudopterosins and *seco*-pseudopterosins isolated from the octocoral *Pseudopterogorgia elisabethae* of San Andrés and Providencia islands (southwest Caribbean Sea), we report the anti-microbial profile against four pathogenic microorganisms (*Staphylococcus aureus*, *Enterococcus faecalis*, *Pseudomonas aeruginosa* and *Candida albicans*) and report a more complete cytotoxic profile against five human cells lines (HeLa, PC-3, HCT116, MCF-7 and BJ) for the compounds PsG, PsP, PsQ, PsS, PsT, PsU, 3-*O*-acetyl-PsU, *seco-*PsJ, *seco-*PsK and IMNGD. For the cytotoxic profiles, all compounds evaluated showed moderate and non-selective activity against both tumor and normal cell lines, where PsQ and PsG were the most active compounds (GI_50_ values between 5.8 μM to 12.0 μM). With respect to their anti-microbial activity the compounds showed good and selective activity against the Gram-positive bacteria, while they did not show activity against the Gram-negative bacterium or yeast. PsU, PsQ, PsS, *seco-*PsK and PsG were the most active compounds (IC_50_ 2.9–4.5 μM) against *S. aureus* and PsG, PsU and *seco*-PsK showed good activity (IC_50_ 3.1–3.8 μM) against *E. faecalis*, comparable to the reference drug vancomycin (4.2 μM).

## 1. Introduction

The discovery of selective and potent therapeutic activity of pseudopterosins and *seco*-pseudopterosins isolated from the octocoral *Pseudopterogorgia elisabethae* [1–14] and the high degree of chemical variation among specimens collected at different locations throughout the Caribbean region [1,2], have motivated several authors to pursue this field of study. Thus far, 30 pseudopterosins (PsA-Y, *iso*-PsE, 2-*O*-Ac-PsQ, 3-*O*-Ac-PsQ, 2-*O*-Ac-PsU and 2-*O*-Ac-PsQ) [3–11] and 11 *seco-*pseudopterosins (*seco-*PsA-K) [8–13] have been isolated from specimens collected in the Bahamas, Bermuda, the Florida Keys and the Colombian islands of San Andrés and Providencia.

The pseudopterosins (PsA-D, PsE, *iso*-PsE, PsM-O, PsX and PsY) and *seco-*pseudopterosins (*seco-*PsA-G) isolated from specimens collected in the North Caribbean Sea (the Bahamas, Bermuda and the Florida Keys) have been evaluated as anti-inflammatory, analgesic and antimicrobial agents [2–6,8,12]. Among them, PsN, PsA, *iso-*PsE and PsE were found to be the most potent compounds in mouse ear anti-inflammatory assays [3,4,6,8]. Furthermore PsA and PsE, appeared to prevent eicosanoid biosynthesis by inhibition of PLA2, 5-LO and COX, degranulation of leukocytes and the consequent liberation of lysosomal enzymes [15,16]. PsA-E, PsK, PsX, PsY and *seco-*PsA-D showed excellent and selective activity against the Gram-positive bacteria *Streptococcus pyogenes*, *Staphylococcus aureus*, and *Enterococcus faecalis* [5,12]. Pseudopterosins are also used in several skin care products [1,17]. Methopterosin is a simple derivative of PsA and has completed Phase I and II clinical trials as a wound healing agent [17–20].

Specimens of *P. elisabethae* collected in the southwest Caribbean Sea, specifically at the Islands of San Andres and Providencia (Colombia), have been shown to contain new pseudopterosins (PsP-V, 2-*O*-Ac-PsQ, 3-*O*-Ac-PsQ, 2-*O*-Ac-PsU and 2-*O*-Ac-PsQ, PsG and PsK), *seco*-pseudopterosins (*seco-*PsH-K), and an inter-converting mixture of non-glycosylated diterpenes (10-acetoxy-9-hydroxy- and 9-acetoxy-10-hydroxy-amphilecta-8,10,12,14-tetraenes (IMNGD)) [7,9–11]. However, only a limited number of these compounds has been evaluated for anti-inflammatory [21], anti-tuberculosis, anti-viral, anti-malarial and anti-cancer [11] activity.

In regards to their anti-inflammatory activity, our experiments showed that fractions enriched with pseudopterosins (PsG, PsK, PsP, PsQ, PsS, PsT and PsU) and *seco*-pseudopterosins (*seco*-PsJ and *seco*-PsK) were able to inhibit the inflammation in a 12-*O*-tetradecanoyl-phorbol-acetate (TPA)-induced edema assay, with results comparable to those shown by indomethacin used as the control standard [21]. Additionally, in *in vitro* experiments, PsQ, PsS, PsT, PsU and IMNGD showed good activity for the inhibition of MPO release; PsP, PsT and IMNGD inhibit NO release [21] and PsR inhibited thromboxane B2 (TXB_2_) and the superoxide anion (O_2_^−^) [11].

In other experiments, PsP was evaluated for anti-viral activity (against HSV-1, HSV-2, HVMV and VZV), and PsQ, PsR, PsU, PsV, *seco-*PsH and *seco-*PsI evaluated as anti-malaria agents (against *Plasmodium falciparum*) and as anti-tuberculosis agents (against *Mycobacterium tuberculosis* H_37_Rv) [11]. There is only one report discussing the cytotoxic activity of PsQ, PsR, PsU, PsV, and 3-*O*-acetyl-PsU against MCF-7 (breast cancer), NCI-H460 (non-small-cell lung cancer), and SF-268 (CNS) cells [11]. These data suggest that some of these pseudopterosins have some cytotoxicity but lack potency.

**Figure 1.**
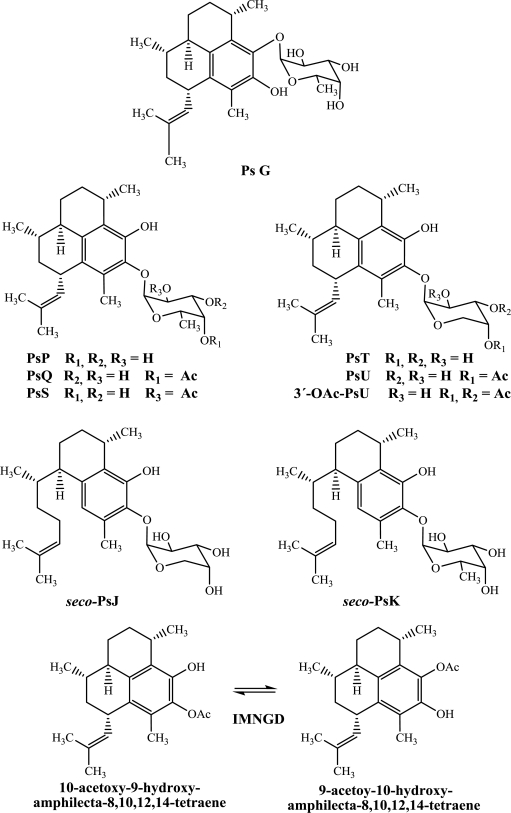
Chemical structures of pseudopterosins, *seco*-pseudopterosins and IMNGD isolated from *P. elisabethae* (SW Caribbean Sea).

While there has been considerable effort towards characterizing the biological activity of pseudopterosins isolated from octocoral samples collected in the northern Caribbean, much is unknown about the bioactivity of such compounds isolated from octocoral collected from the Islands of San Andrés and Providencia. For this reason, and with a goal of expanding the potential of these compounds, here we report for the first time the antimicrobial profile against four pathogen microorganisms (*S. aureus*, *E. faecalis*, *Pseudomonas aeruginosa* and *Candida albicans*) and report a more complete cytotoxic profile against five human cells lines (HeLa, PC-3, HCT116, MCF-7 and BJ) for the compounds: PsG, PsP, PsQ, PsS, PsT, PsU, 3-*O*-acetyl-PsU, *seco-*PsJ, *seco-*PsK and IMNGD (Figure 1).

## 2. Results and Discussion

In this study, PsG, PsP, PsQ, PsS, PsT, PsU, 3-*O*-acetyl-PsU, *seco-*PsJ, *seco-*PsK and IMNGD were isolated (Figure 1) by flash chromatography and HPLC and identified by spectroscopic means. The structures of all compounds were previously reported by us [7,10], and preliminary assessment of their cytotoxic (Table 1) and antimicrobial (Table 2) properties is presented here.

### 2.1. Cytotoxic Activity

The cytotoxic activity of the pseudopterosins (PsG, PsP, PsQ, PsS, PsT, PsU and 3-*O*-acetyl-PsU), *seco*-pseudopterosins (*seco-*PsJ and *seco-*PsK) and IMNGD against five cell lines: HeLa (cervical cancer), PC-3 (prostate cancer), HCT116 (colorectal cancer), MCF-7 (breast cancer) and BJ (fibroblasts) was investigated using the MTT reduction colorimetric assay [22]. Although no compound showed significant activity, according to the GI_50_ cut off value of 10 nM for pure compounds suggested by Bugelski *et al.* [23], all compounds evaluated showed moderate to weak (GI_50_ 5.8–83.9 μM) and non-selective activity against both tumor and normal cell lines as shown in Table 1.

While none of these compound showed comparable activity to the reference drug staurosporine (GI_50_ 13.6–105.6 nM) against the four tumor cell lines, PsQ and PsG were the most active compounds (GI_50_ values between 5.8 μM to 12.0 μM) and IMNGD showed a moderate activity with GI_50_ values of 9.7–19.9 μg/mL. These results are comparable with those previously reported by Rodriguez *et al.* [11], who determined the GI_50_ values for PsQ, PsU and PsV in the NCI-H460-cell line. Results for PsQ showed a GI_50_ between 1.7–5.8 μM. In the same screen, PsU and PsV were generally much less toxic (GI_50_ 20–100 μM). For the normal cell line BJ (Table 1), PsS, 3-*O*-acetyl-PsU, PsP, PsT and *seco*-PsJ did not show a considerable cytotoxic effect (GI_50_ > 10 μM) while PsQ, PsG, *seco*-PsK and PsU showed a moderate cytotoxic activity (GI_50_ 4.5–9.3 μM). The IMNGD showed a moderate activity with GI_50_ of 8.3 μg/mL.

The selectivity of the cytotoxic activity of the compounds, measured as the differential effect on growth of different types of cell lines, was made by comparing the effect of the compounds on inhibiting cell growth in both normal and tumor cells. The results showed no selectivity of compounds between the lines used, and all compounds induced reduction in cell survival to a similar magnitude in all lines.

Preliminary conclusions regarding structure-activity relationships can be drawn from an examination of the cytotoxic activity. The position of glycosylation on the terpene skeleton appears to affect the inhibitory activity profile as, for example, PsG (glycosylated in C-9 with fucopyranose) is more active than PsP (glycosylated in C-10 with fucopyranose). Further, the type of sugar moiety also influences the activity as, for example, PsP which is glycosylated with fucopyranose is more active than PsT which is glycosylated with arabinopyranose. Likewise, PsQ (C-4’ mono-acetylated fucose as sugar moiety) is more active than PsU (C-4’ mono-acetylated arabinose as sugar moiety) and *seco*-PsK (non-acetylated fucose as sugar moiety) is more active than *seco*-PsJ (mono-acetylated arabinose as sugar moiety).

### 2.2. Antimicrobial Activity

The antimicrobial activity of the pseudopterosins, *seco*-pseudopterosins and IMNGD was investigated against one Gram-negative bacterium (*P. aeruginosa*), two Gram-positive bacteria (*S. aureus* and *E. faecalis*) and one yeast (*C. albicans*), using a microdilution method [24]. The majority of compounds evaluated showed a good IC_50_ (2.9–7.64 μM) and all showed a selective activity against both Gram-positive bacteria tested (Table 2), while they did not show activity against the Gram-negative bacterium or the yeast.

PsU, PsQ, PsS, *seco-*PsK, PsG were the most active compounds (IC_50_ 2.3–4.5 μM) against *S. aureus*. The IMNGD showed good activity with IC_50_ of 2.3 μg/mL. For *E. faecalis*, PsG, PsU and *seco*-PsK showed better activity (Table 2) compared to the reference drug vancomycin (4.2 μM) while, *seco*-PsJ and PsT exhibited similar activity. The IMNGD showed good activity, with an IC_50_ of 3.5 μg/mL. These results are comparable with the activity previously reported for PsA-E, PsK, PsX and PsY isolated from specimens collected in the north Caribbean Sea [5], which were reported to have minimum inhibitory concentration (MIC) values between 4.2 and 8.8 μM.

The selectivity observed in our study is comparable to that reported by Ata *et al.*, [5] who reported that other pseudopterosins (PsA-E, PsK, PsX and PsY) inhibit the growth of Gram-positive bacteria (*S. pyogenes*, *S. aureus*,and *E. faecalis*), while they were inactive against Gram-negative bacteria (*E. coli* and *P. aeruginosa*). This finding can be related to the fact that many Gram-negative bacteria are resistant to toxic agents in the environment, due to the barrier of lipopolysaccharides on their outer membrane [25].

Examination of the data in Table 2 suggests the following structure–activity relationships. Firstly, PsG (glycosylated in C-9 with fucopyranose) is more active than PsP (glycosylated in C-10 with fucopyranose). Secondly, PsT glycosylated with arabinopyranose is more active than PsP, which is glycosylated with fucopyranose. Likewise, PsU (mono-acetylated arabinose as sugar moiety) is more active than PsQ and PsS (mono-acetylated fucose as sugar moiety). However, this behavior initially observed in pseudopterosins (amphilectane skeleton) is not retained when the results are compared with *seco*-psedopterosins (serrulatane skeleton), where, the *seco*-PsK (glycosylated with fucoyranose) showed more activity than *seco*-PsJ (glycosylated with arabinopyranose). Clearly, additional work is required to fully understand the structure–activity relationships for this family of terpenes.

From the above discussion, one can note that the pseudopterosins, *seco-*pseudopterosins and IMNGD isolated from *P. elisabethae* collected at the Islands of San Andrés and Providencia (southwest Caribbean Sea) have a similar drug-like potential as the related compounds isolated from specimens collected in the north Caribbean Sea (the Bahamas, Bermuda and the Florida Keys). Further, due to their wide applicability in commercial additives, in the cosmetic industry, it might be important to consider *P. elisabethae* collected in Colombian waters as an alternative source of such compounds. As has been shown, the anti-inflammatory, anti-microbial and cytotoxic activity of this family of compounds isolated from *P. elisabethae* collected at different locations at the Caribbean Sea are quite similar, regardless of a small number of differences in their structure (type of sugar moiety, position of glycosylation and stereochemistry).

## 3. Experimental Section

### 3.1. Chemicals and Reagents

The following substances were purchased from Sigma-Aldrich (St Louis, USA): Dulbecco’s modified Eagle’s medium (DMEM), 3-[4,5-dimethylthiazol-2-yl]-2,5-diphenyltetrazolium bromide (MTT), staurosporine, vancomycin, penicillin G, gentamicin, nystatin, Luria-Bertani Broth (LB) and Sabouraud Dextrose broth (SD). Fetal bovine serum (FBS), salts for phosphate buffered saline (PBS) solution and organic solvents, were purchased from WRW (PA, USA).

### 3.2. Octocoral Collection

Fragments of individual colonies of *P. elisabethae* were collected by SCUBA (*ca*. 20–30 m depth) at Providencia and San Andrés islands (SW Caribbean) and identified by Dr. Monica Puyana. Voucher specimens coded as INV CNI 1612–1616, were deposited at the invertebrate collection of Museo de Historia Natural Marina Colombiana (MHNMC) at Instituto de Investigaciones Marinas de Punta Betín (INVEMAR).

### 3.3. Isolation and Structure Elucidation of Compounds from *P. elisabethae*

The dried colony fragments (30 g) from each location were extracted separately with a dichloromethane-methanol (1:1) mixture. The resulting extracts were filtered and concentrated by rotary evaporation to obtain a dark green oil. The isolation of each compound was carried out according to our previously described procedures [7,10] with some modifications. Each extract was subjected separately to flash C18 column chromatography and eluted with 500 mL of each solvent mixture of decreasing polarity (methanol-water 1:9, fraction **F1**; methanol-water 1:1, **F2**; methanol-water 4:1, **F3**; methanol 100% **F4**; ethanol 100% 5, **F5**; acetone 100%, **F6**; dichloromethane-methanol 5:5, **F7;** and dichloromethane 100%, **F8)**. Checking by LC-MS and TLC, the fractions **F4** and **F5** from the Providencia extract contained a mixture of pseudopterosins and *seco*-pseudopterosins and **F5** from the San Andrés extract contained the interconverting mixture of non-glycosylated diterpenes (IMNGD). Fraction **F4** and **F5** from Providencia were mixed and subsequently separated by flash chromatography using a diol column (150 g) yielding PsG (19.1 mg), PsP (9.6 mg), PsQ (58.1 mg), PsS (2.3 mg), PsT (14.5 mg), PsU (46.6 mg), 3-*O*-Ac-PsU (3.5 mg), *seco*-PsJ (16.2 mg) and *seco*-PsK (1.2 mg). Final purification of all compounds was performed on HPLC, using a column Gemini C-18 (5 μm, 10 × 250 mm) and MeOH-water (9:1) as the mobile phase with a 3.0 mL/min flow rate. The isolated compounds were carefully identified by comparison of their spectral data with that shown by our previously isolated compounds [7,8]. Purity was confirmed by HPLC and NMR.

### 3.4. Cell Lines

The cell lines HeLa (cervical cancer), PC-3 (prostate cancer), HCT116 (colorectal cancer), MCF-7 (breast cancer) and normal human cell line BJ (skin fibroblasts) were used and maintained in DMEM with 5% of FBS and gentamicin 50 μg/mL. Cultures were held in 75 cm^2^ culture flasks at 37 °C, 5% CO_2_ and 100% relative humidity, changing media at least twice a week.

### 3.5. Cytotoxic Assay

Cells were harvested, counted, and transferred into 96 well plates and incubated for 24 h prior to the addition of the test compounds. Test sample solutions (10 μL) at the desired dilutions were added to the wells containing the cells and incubated for 48 h. Microtitration colorimetric method of MTT reduction was used to determine surviving cells at the end of the treatment period [22].

A solution of MTT (0.5 mg/mL) was prepared in PBS and 100 μL of this solution was added to each well after the removal of 100 μL of treatment solution. The plates were incubated at 37 °C for 4 h. The solution in each well, containing media, unmetabolized MTT, and dead cells, was removed by suction and 100 μL of DMSO was added to each well. The DMSO was mixed by pipetting and optical density was recorded using a BioTek Synergy HT microplate reader to measure the absorbance at 570 nm. Percentages of cell survival relative to vehicle control wells (wells containing only cells and DMSO) were calculated.

Stock solutions (5 mg/mL) were prepared by dissolving pure compounds in DMSO and storing at 4 °C. Serial dilutions with culture media were prepared just prior to addition to test plates. The concentration of DMSO in wells was consistent across dilutions at 0.38% v/v. Staurosporine was used as the positive control and vehicle (DMSO + media) as blank. Control and test compounds (PsG, PsP, PsQ, PsS, PsT, PsU, 3-O-Ac-PsU, *seco*-PsJ, *seco*-PsK and IMNGD) were assayed in duplicate for each concentration and replicated for each cell line at concentrations of 5000, 1000, 500, 100, 50, 10, 5 and 1 nM for staurosporine and 0.1, 0.5, 1.0, 2.5, 5.0, 7.5, 10.0, and 20.0 μg/mL for compounds.

### 3.6. Microbial Strains and Culture Media

Compounds isolated from *P. elisabethae* were tested against a panel of microorganisms including the bacteria *Staphylococcus aureus* ATCC 375, *Enterococcus faecalis* ATCC 10741, *Pseudomonas aeruginosa* ATCC 14210 and the yeast *Candida albicans* ATCC 14035. Bacterial strains were cultured in Luria-Bertani Broth (LB) and *C. albicans* was cultured in Sabouraud dextrose broth (SD), for 18 h at 37 °C and 220 rpm in a humidified incubator.

### 3.7. Antimicrobial Assay

The antimicrobial activity was determined by a microdilution method (IC_50_) in 96-well microtiter plates using LB media for bacteria and SD media for the yeast seeded liquid media [24]. In all cases, a pre-inoculated dilution was made with fresh broth. A volume of 180 μL of each microorganism suspension was inoculated in each well (10^5^ cells/mL) and mixed with 20 μL of treatment solutions. Stock solutions were prepared by dissolving pure compounds and controls in 20% DMSO and storing at 4 °C. Penicillin G *(S. aureus*), vancomycin (*E. faecalis*), gentamicin (*P. aeruginosa*) and nystatin (*C. albicans*) were used as positive controls and vehicle (DMSO + media) as blank. The final DMSO concentration used for dissolving the extracts was less than 20%. Controls and test compounds (PsG, PsP, PsQ, PsS, PsT, PsU, 3-O-Ac-PsU, *seco*-PsJ, *seco*-PsK and IMNGD) were assayed in duplicate for each of the following concentrations: 64.0, 32.0, 16.0, 4.0, 1.0, 0.25 and 0.06 μg/mL.

Microplates were incubated for 24 h at 37 °C and optical density was recorded using a BioTek Synergy HT microplate reader to measure the absorbance at 600 nm. For each experiment, correction for background absorbance was made by subtracting the value for OD_600_ after the compounds were added (time 0 h). Percentages of microorganism survival relative to vehicle control wells (wells containing only microorganism and DMSO) were calculated. The IC_50_ of the controls were also determined in parallel experiments in order to control for the sensitivity of the standard test organisms.

### 3.8. Statistical Analysis

Data were normalized and analyzed using non-linear regression to elucidate IC_50_ values from sigmoidal dose-response curves. Comparison between cell lines and microorganisms used extra sum-of-squares F test to evaluate IC_50_ between data sets. P < 0.05 was considered as indicative of significance using GraphPad Software, Prism V. 5.0.

## 4. Conclusions

All results presented here contribute to the demonstration that the compounds isolated from *P. elisabethae* (SW Caribbean) are promising molecules with potentially useful antimicrobial activity profiles. This confirms that this marine organism has great value as a source of lead compounds with pharmaceutical applications. Specifically, PsU, PsQ, PsS, *seco-*PsK and PsG are the most promising anti-bacterial agents. For this reason, it will be interesting to continue with molecular targeted high-throughput screens in order to understand their mechanism of action.

## Figures and Tables

**Table 1.** Cytotoxic effect of diterpenes isolated from *P. elisabethae* (SW Caribbean) in human cancer cell lines (HeLa, PC-3, HCT116, MCF-7) and normal BJ cells.

**Compounds**	**GI_50_ ± S.E (μM)**				
	**HeLa**	**PC-3**	**HCT116**	**MCF7**	**BJ**
PsG	9.22 ± 0.45	8.83 ± 0.54	12.04 ± 0.36	9.42 ± 0.43	7.62 ± 0.38
PsP	10.31 ± 0.49	13.77 ± 0.58	17.89 ± 0.45	12.58 ± 0.45	10.40 ± 0.42
PsQ	5.82 ± 0.33	7.81 ± 0.35	7.66 ± 0.27	8.44 ± 0.41	4.47 ± 0.31
PsS	13.79 ± 0.39	52.05 ± 0.31	33.50 ± 0.33	26.25 ± 0.45	29.14 ± 0.45
PsT	14.58 ± 0.35	21.99 ± 0.46	24.24 ± 0.42	14.72 ± 0.46	12.94 ± 0.37
PsU	15.63 ± 0.32	24.81 ± 0.34	23.44 ± 0.40	26.46 ± 0.46	9.35 ± 0.34
3-*O*-Ac-PsU	44.61 ± 0.35	26.45 ± 0.31	20.48 ± 0.48	83.93 ± 0.39	62.03 ± 0.45
*seco*-PsJ	21.08 ± 0.37	37.21 ± 0.41	31.68 ± 0.32	28.02 ± 0.51	15.00 ± 0.35
*seco*-PsK	15.83 ± 0.18	13.57 ± 0.38	13.28 ± 0.27	11.45 ± 0.36	8.28 ± 0.29
IMNGD^*^	11.20 ± 0.15	12.11 ± 0.20	19.90 ± 0.19	9.67 ± 0.15	7.91 ± 0.17
Staurosporine^**^	105.6 ± 0.41	61.82 ± 0.38	45.56 ± 0.45	176.6 ± 0.38	13.56 ± 0.35

* GI_50_ (μg/mL),

** GI_50_ (nM)

**Table 2.** Antibacterial activity of diterpenes isolated from *P. elisabethae* (SW Caribbean) in two Gram-positive bacteria.

**Compounds**	IC_50_ ± S.E (μM) *	
	***S. aureus***	***E. faecalis***
PsG	4.48 ± 0.18	3.14 ± 0.22
PsP	14.91 ± 0.20	37.35 ± 0.29
PsQ	3.30 ± 0.20	7.38 ± 0.16
PsS	3.89 ± 0.23	20.20 ± 0.25
PsT	5.39 ± 0.25	4.38 ± 0.16
PsU	2.97 ± 0.17	3.19 ± 0.25
3-*O*-Ac-PsU	20.23 ± 0.19	7.64 ± 0.16
*seco*-PsJ	6.52 ± 0.12	4.08 ± 0.19
*seco*-PsK	4.20 ± 0.16	3.82 ± 0.21
IMNGD^**^	2.33 ± 0.09	3.47 ± 0.15
Penicillin G	1.61 ± 0.08	-
Vancomycin	-	4.21 ± 0.11

* All compounds evaluated were inactive against the Gram-negative bacterium *P. aeruginosa* and the yeast *C. albicans*.

** IC_50_ (μg/mL).
